# Migration of the uncemented Echo Bi-Metric and Bi-Metric THA stems: a randomized controlled RSA study involving 62 patients with 24-month follow-up

**DOI:** 10.1080/17453674.2020.1802682

**Published:** 2020-08-06

**Authors:** Karen Dyreborg, Mikkel R Andersen, Nikolaj Winther, Søren Solgaard, Gunnar Flivik, Michael M Petersen

**Affiliations:** a Department of Orthopaedic Surgery, Rigshospitalet, University of Copenhagen, Denmark; b Department of Hip and Knee Surgery, Herlev-Gentofte University Hospital, Hellerup, Denmark; c Department of Orthopedics, Lund University and Skåne University Hospital, Lund, Sweden

## Abstract

Background and purpose — Despite the good results after total hip arthroplasty (THA), new implants are continuously being developed to improve durability. The Echo Bi-Metric (EBM) THA stem is the successor to the Bi-Metric (BM) THA stem. The EBM stem includes many of the features of the BM stem, but minor changes in the design might improve the clinical performance. We compared the migration behavior with radiostereometric analysis (RSA) of the EBM stem and the BM stem at 24 months and evaluated the clinical outcome.

Patients and methods — We randomized 62 patients with osteoarthritis (mean age 64 years, female/male 28/34) scheduled for an uncemented THA to receive either an EBM or a BM THA stem. We performed RSA within 1 week after surgery and at 3, 6, 12, and 24 months. The clinical outcome was evaluated using Harris Hip Score (HHS) and Oxford Hip Score (OHS).

Results — At 24 months, we found no statistically significant differences in migration between the two implants. During the first 3 months both the EBM and the BM stems showed visible subsidence (2.5 mm and 2.2 mm respectively), and retroversion (2.5° and 2.2° respectively), but after 3 months this stabilized. The expected increase in HHS and OHS was similar between the groups.

Interpretation — The EBM stem showed a migration at 24 months not different from the BM stem, and both stems display satisfying clinical results.

To improve the longevity of total hip arthroplasty (THA) new designs are continuously being developed. The introduction of new implants should optimally be done by phased stepwise introduction (Malchau 1995, Nelissen et al. 2011) including radiostereometric analysis (RSA) of implant migration.

Some subsidence of hip stems is generally accepted within the first 3 months, but after that osseointegration and stability should have occurred. Mean subsidence of up to 1 mm of the stem at 24 months has been reported (Nysted et al. 2014, Weber et al. 2014, Hoornenborg et al. 2018, Sesselmann et al. 2018, Kruijntjens et al. 2020).

This study investigates by RSA potential differences in migration at 24 months, between 2 different designs of porous-coated uncemented hip prosthesis; the Bi-Metric Full Proximal Profile THA stem (BM) and the Echo Bi-Metric (EBM) stem (Zimmer Biomet, Warsaw, IN, USA) (Figure 1). Both stems are press-fit titanium alloy stems with a proximal plasma spray porous titanium coating and a distal part with a roughened titanium surface. The BM has shown good clinical results and excellent stem survival in register studies since its introduction in 1984 (Jacobsen et al. 2003, Davies et al. 2010, Mäkelä et al. 2010, Lazarinis et al. 2011). The EBM is the successor to the BM and has 3 theoretical design improvements: a slimmer design of the neck to increase range of motion; a polished bullet-shaped distal tip to reduce distal stress; and an extended porous coating to support biological ingrowth proximally. Evaluation of adaptive bone remodeling and stress shielding will be addressed in another publication.

**Figure 1.**
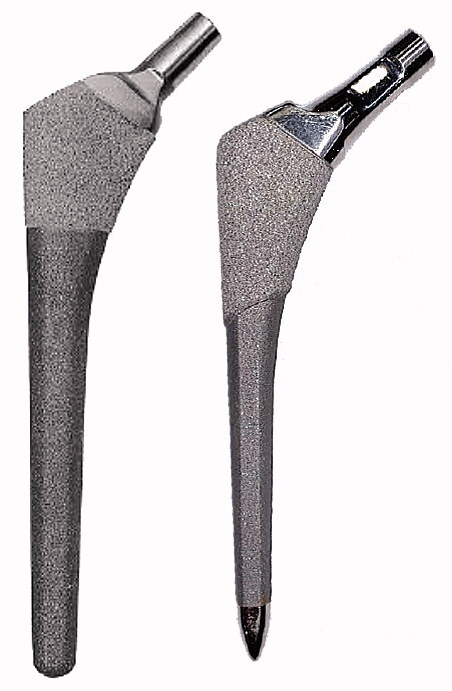
The stems: on the left the Bi-Metric stem and on the right the Echo Bi-Metric stem.

We hypothesized that the migration of the EBM was less at 24 months, compared with the BM stem.

## ^Patients and methods^

### ^Design and participants^

This study is a randomized controlled trial, allocation ratio 1:1.

The inclusion criteria were: patients with primary osteoarthritis scheduled to undergo THA at the Herlev-Gentofte University Hospital, Department of Hip and Knee Surgery, age 30–75 years, and informed consent. The exclusion criteria were: infection, diseases affecting the bone metabolism (osteoporosis, osteomalacia, Paget’s disease, hypo- or hyperparathyroidism, vitamin D deficiencies, cancer, avascular necrosis, or rheumatoid arthritis), pregnancy, inability to cooperate, inability to communicate in Danish, and medicine or alcohol abuse. The secondary exclusion criterion was too few markers visible.

### ^Randomization and blinding^

Prior to study start the randomization code was generated by a web-based program and envelopes with the individual allocation were sequentially numbered. The allocation was performed as block randomization with blocks of 10 and the sequence was locked away. The randomization allocation sequence and packaging of non-transparent and closed envelopes was done by a colleague outside the project. Screening and enrollment were done by the primary investigator (KD). When the patient was ready in the operating theater, the numbered envelope was opened.

Due to visual differences of the 2 prostheses, the surgeon and health personnel were not blinded. The participants were all blinded.

### ^Surgery^

All patients received either an uncemented EBM or BM THA stem. A 32 mm CoCr head and an Exceed ABT RingLoc-x acetabular shell (Zimmer Biomet) with a highly cross-linked polyethylene liner were implanted in all patients.

Surgery was performed with a posterolateral approach by 1 of 4 experienced hip surgeons. Prior to each procedure and allocation, the surgeons templated the stems on calibrated radiographs to anticipate the size and position of the stem and cup. The stems do not differ in length, diameter, or offset for a given size.

Surgery was performed under spinal or general anesthesia. On the day of surgery, the patients were mobilized with full weight-bearing using crutches and physiotherapy began. All patients were given oral anticoagulants (rivaroxaban until discharge), and prophylactic antibiotics (dicloxacillin, 2 g preoperatively and 1 g x 2 postoperatively) during the first 24 hours.

### ^RSA^

**Figure 2.**
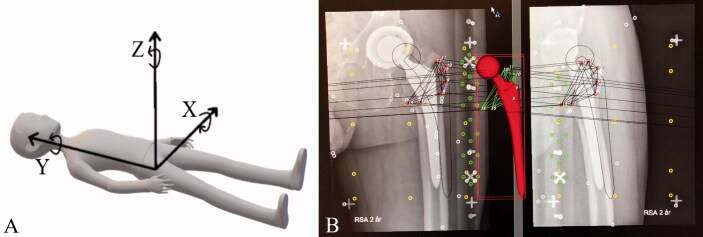
(A) The RSA coordinate system with movements; (B) model-based RSA screenshot while using the RSAcore software.

During surgery 8 to 10 tantalum markers (0.8 mm) were inserted in a well-scattered manner into trochanter minor and trochanter major, respectively. Instead of markers on the stems we used CAD models from the RSA software manufacturer with an added 3D surface model of the spherical head as described by Prins et al. (2008), giving us the possibility of model based-RSA (MB-RSA). Within 1 week of discharge the patients had their baseline RSA radiographs taken at the Department of Diagnostic Radiology at Rigshospitalet, Copenhagen, Denmark (mean days from surgery: BM = 7 and EBM = 6). The required set up with 2 ceiling-mounted X-ray tubes was used with a uniplanar calibration cage (RSA Biomedical cage 41), defining the coordinate system, to take 2 simultaneous digital radiographs at an angle of 42° apart. The patients were placed in a supine position and the operated limb fixed in maximum external rotation to visualize as much as possible of the lesser trochanter (Figure 2). The follow-up examinations were scheduled at 3, 6, 12, and 24 months. Migration of the stems was calculated using CAD models in the model-based RSA software (version 4.1; RSAcore, Department of Orthopaedic Surgery, LUMC, the Netherlands) at the Biomechanics and RSA laboratory at Skåne University Hospital, Lund, Sweden. Migrations for left-sided prostheses were recalculated, making all results right-handed.

55 double examinations were performed with total repositioning of the patients, to estimate the precision of the RSA set-up, i.e., the random deviation. Precision error (PE), defined as 2 standard deviations, was calculated for the rotational and translational segment motions. PE for X-, Y-, and Z-translation was 0.15 mm, 0.27 mm, and 0.54 mm, respectively. For the X-, Y-, and Z-rotation PE was 0.82°, 2.32°, and 0.25°, respectively.

Mean error of rigid body fitting was limited to 0.35 mm. The condition number limit was set at 150.

### ^Clinical outcome^

We used Harris Hip Score (HHS) and Oxford Hip Score (OHS) (Fitzpatrick et al. 2007, Paulsen et al. 2012) preoperatively and at 6, 12, and 24 months to evaluate the clinical outcome.

### ^Outcomes^

The primary outcome measure was Y-translation at 24 months.

Secondary outcome measures were (1) Y-rotation, X- and Z-movements at the time intervals 3, 6, 12, and 24 months and (2) clinical outcome, monitored with HHS and OHS (postoperatively and at 6, 12, and 24 months). The minimally important difference estimate for HHS is 18 (Singh et al. 2016); for OHS it is 5 (Beard et al. 2015).

### ^Sample size^

Our power analysis was based on the standard deviation (SD) for the migration after 2 years of follow-up from 2 previously published studies, with information regarding SD (Ström et al. 2006, Wierer et al. 2013). Hence, our SD was 0.69 mm, our minimal relevant difference (MIREDIF) = 0.6 mm, type I error = 5%, and type II error = 15% (resulting in a standardized difference of 0.87). Aiming for a power of 80–90% required a minimum of 23 hips in each group. We included 31 in each group to accommodate future dropouts.

### ^Statistics^

Data were tested for normality by histogram, QQ plot, and Kolmogorov–Smirnov, and the data were not normal distributed. For evaluation of potential differences between the mean migrations, we used a Mann–Whitney U-test.

All data are presented as mean (SD) unless otherwise reported. The data on the secondary outcomes were considered as being exploratory in nature, and were adjusted for multiplicity only if statistically significant differences (p < 0.05) were found. 95% confidence intervals (CI) were calculated for RSA data.

The statistical software SPSS version 24 (IBM Corp, Armonk, NY, USA) was used.

### ^Ethics, registration, funding, and potential conflicts of interest^

Written informed consent was obtained from all the subjects before enrollment.

This RCT was approved by the local Regional Ethics Committee (H-4-2014-079) and by the Danish Data Protection Agency (GEH-2015-079, I-Suite no. 03764) and registered at ClinicalTrials.gov (NCT02656771) prior to enrollment as a combined registration for the present study and a study of adaptive bone remodeling (not yet published). We have not changed the endpoints after trial initiation; however, we have specified our primary outcome after trial initiation, since its formulation was too imprecise (Evans 2007). The study was carried out in accordance with the principles of the Helsinki Declaration, and data are presented following the CONSORT statement and The guidelines for standardisation of radiostereometry of implants (Valstar et al. 2005). The study was partly funded by Zimmer Biomet (grant number C004287X). Zimmer Biomet had no access to data or impact on the data interpretation. MMP reports grants from Zimmer Biomet during the conduct of the study. All other authors have no conflict of interests related to the manuscript.

## ^Results^

**Figure 3.**
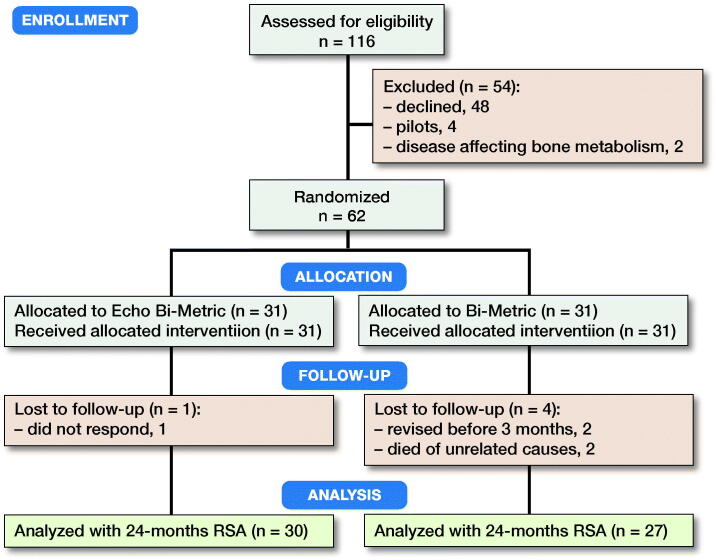
Flowchart

From February 2016 to September 2017 we screened 116 patients, enrolled and randomized 62 patients (mean age = 64 years [49–74], female/male = 28/34) to receive either an EBM (n = 31) or a BM (n = 31) THA stem (Figure 3). The distribution of THAs among the 4 surgeons was 5, 6, 23, and 28 patients, respectively. In the EBM group, 1 patient was lost to follow-up for unknown reasons (did not respond to contact attempts). In the BM group, 4 patients were lost to follow-up; 2 were revised before 3 months (1 periprosthetic fracture and 1 undersized stem) and 2 died of causes unrelated to the surgery (Figure 3, Table 1).

**Table 1.** Baseline demographics. Values are mean (range) unless otherwise specified

	Bi-Metric	Echo Bi-Metric
Age, years	66 (49–74)	63 (50–74)
Sex, male/female, n	17/14	17/14
Height, m	1.77 (1.60–1.96)	1.76 (1.60–1.91)
Weight, kg	84 (50–124)	83 (54–122)
BMI	27 (18–38)	27 (20–36)
Operated side, R/L, n	18/13	15/16
Cup size	56 (50–62)	56 (50–62)
Stem size	12 (9–16)	11 (7–16)

### ^Primary outcome^

**Figure 4.**
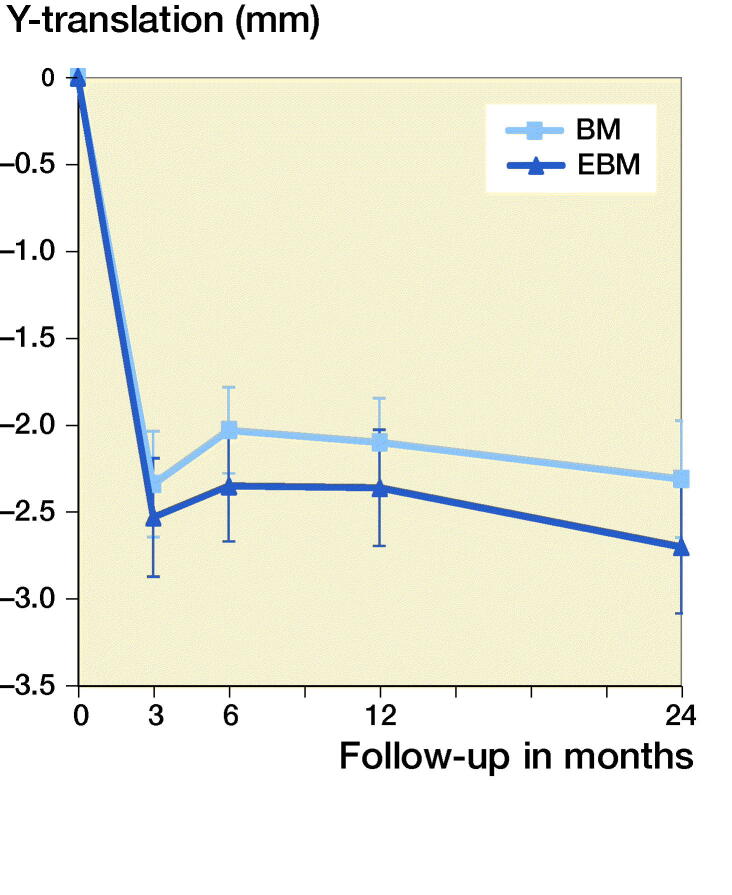
Mean Y-translation at each follow-up for the 2 stems (bars are standard error of mean).

Subsidence (negative Y-translation) at 24 months for the BM was 2.3 mm, for EBM 2.7 mm (p = 0.6). For both the BM and the EBM initial subsidence was seen up to 3 months (Table 2, Figure 4). At 3 months the stems had subsided 2.5 mm for the EMB and 2.2 mm for the BM stem with no statistically significant differences between the groups at 3 months or at any of the measuring points from 3 to 24 months. There were no statistical outliers regarding Y-translation (outliers as defined by SPSS).

**Table 2.** Mean segment motion (95% CI) of the Bi-Metric (BM) and the Echo Bi-Metric (EBM) stems at follow-up until 24 months. Outliers are included in the analysis

	3 months	6 months	12 months	24 months
BM/EBM, n	29/31	29/31	28/31	27/30
Stem translation, mm				
Y–translation: proximal (+), distal (–)				
BM	–2.2 (–2.3 to –1.7)	–2.0 (–2.5 to –1.6)	–2.1 (–2.6 to –1.6)	–2.3 (–3.0 to –1.7)
EBM	–2.5 (–3.2 to –1.9)	–2.4 (–3.0 to –1.7)	–2.4 (–3.0 to –1.7)	–2.7 (–3.5 to –2.0)
X–translation				
BM	–1.1 (–0.5 to –0.1)	–0.4 (–0.7 to –0.1)	0.1 (–0.3 to 0.0)	–0.4 (–0.6 to –0.1)
EBM	–0.5 (–0.7 to –0.3)	–0.6 (–0.9 to –0.4)	–0.3 (–0.4 to –0.1)	–0.5 (–0.8 to –0.3)
Z–translation				
BM	–0.8 (–1.0 to –0.5)	–0.5 (–0.7 to –0.3)	–0.9 (–1.2 to –0.6)	–0.7 (–1.1 to –0.4)
EBM	–0.8 (–1.0 to –0.6)	–0.4 (–0.6 to –0.2)	–0.9 (–1.2 to –0.7)	–0.9 (–1.1 to –0.6)
Stem rotation, degrees				
Y–rotation: anteversion (–), retroversion (+)				
BM	2.2 (1.5 to 5.0)	2.4 (1.7 to 3.2)	2.5 (1.7 to 3.2)	2.5 (1.6 to 3.3)
EBM	2.5 (1.8 to 3.5)	2.6 (1.9 to 3.5)	2.7 (1.9 to 3.7)	2.7 (1.9 to 3.8)
X–rotation				
BM	–0.6 (–0.9 to –0.3)	–0.2 (–0.5 to 0.1)	–0.6 (–1.0 to –0.2)	1.2 (0.7 to 1.6)
EBM	–0.4 (–0.8 to –0.03)	0.0 (–0.2 to 0.3)	–0.8 (–1.2 to –0.5)	1.1 (0.7 to 1.6)
Z–rotation: varus (–), valgus (+)				
BM	1.7 (1.0 to 2.5)	1.7 (0.9 to 2.5)	1.8 (1.0 to 2.7)	2.0 (1.1 to 2.8)
EBM	1.7 (0.9 to 2.4)	1.8 (1.0 to 2.5)	1.8 (1.1 to 2.6)	2.0 (1.2 to 2.8)

### ^Secondary outcomes^

**Figure 5.**
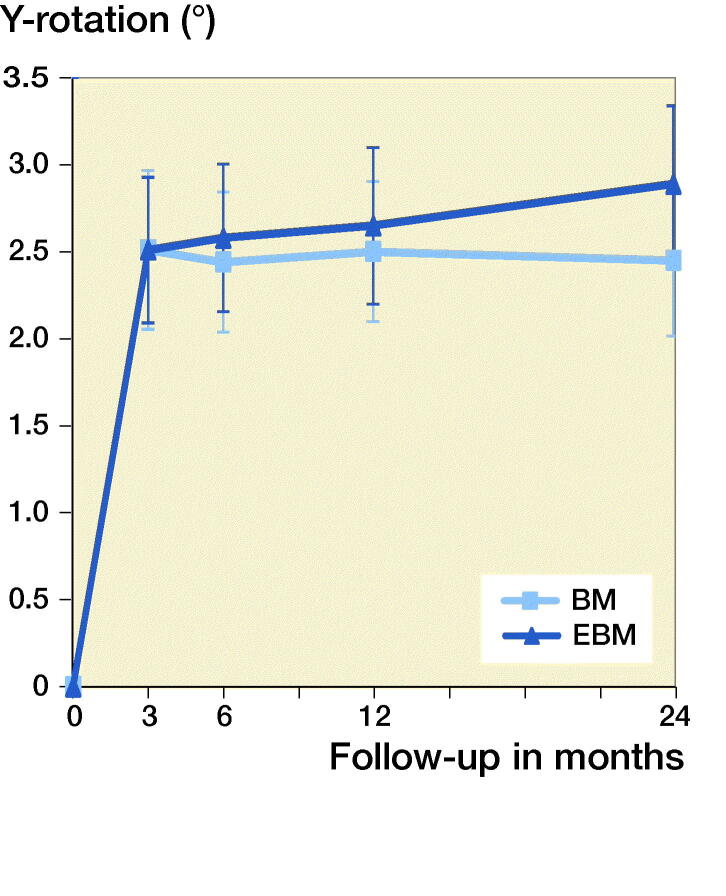
Mean Y-rotation at each follow-up for the 2 stems (bars are standard error of mean).

The ante-retroversion (Y-rotation) was similar between the groups: from 0–3 months retroversion of 2.2° for the BM and 2.5° for the EBM stem was observed and then both stem types stabilized (Table 2, Figure 5).

From 3 to 24 months we found 3 consequent outliers in Y-rotation (Table 3).

**Table 3.** Y-rotation (°) outliers and and their 24-month clinical score

ID (group)	3 months	6 months	12 months	24 months	OHS	HHS
6 (EBM)	12.8	13.4	14.0	14.3	48	100
8 (BM)	6.9	7.0	7.1	8.0	43	100
23 (BM)	–3.3	–4.2	–4.8	–5.2	48	100

The varus–valgus tilt (Z-rotation) was similar for both groups, with a little valgus tilt (at 24 months BM = 2.0°, EBM = 2.0°) (Table 2).

The anterior/posterior tilt (X-rotation) as well as X- and Z-translation stabilized after 3 months (Table 2).

HHS and OHS increased similarly in both groups (Table 4).

**Table 4.** Clinical outcome. Values are mean (range)

Score	BM	EBM	p-value
HSS			
Preoperative	61 (38–82)	67 (31–85)	0.1
6 months	92 (61–100)	91 (70–100)	0.2
12 months	97 (81–100)	95 (73–100)	1.0
24 months	99 (91–11)	98 (49–100)	0.6
OHS			
Preoperative	24 (10–35)	23 (9–40)	0.7
6 months	43 (29–48)	44 (27–48)	0.5
12 months	46 (31–48)	44 (26–48)	0.3
24 months	47 (43–48)	46 (21–48)	0.9

## ^Discussion^

In this RCT comparing the EBM with the BM, we found no statistically significant differences in migration or clinical outcome.

Some degree of stem subsidence and retroversion of uncemented hip prostheses has previously been reported and was confirmed by our study. The stems subsided approximately 2.5–3 mm at 24 months. Osseointegration can be assumed if an RSA study shows stable migration values after 3 months. Nevertheless, the subsidence was higher than reported in other recent studies, where Y-translation is between –0.03 mm and –0.99 mm at 24 months (Nysted et al. 2014, Weber et al. 2014, Nebergall et al. 2016, Mahmoud et al. 2017, Hoornenborg et al. 2018, Sesselmann et al. 2018, Klein et al. 2019, Kruijntjens et al. 2020). Nebergall et al. (2016) (the Taperloc stem), reported a single outlier of –0.9 mm with the rest of the cohort in a range of –0.1 mm to –0.2 mm. Hence, they found more than 10 times less migration compared with our findings. However, their median time from operation to first follow-up was 14 days (0–43), which makes their “postoperative value” closer to the “value at stabilization.”

In the study by Søballe et al. (1993) of the Bi-Metric with or without hydroxyapatite (HA) coating, a subsidence of 0.07–0.09 mm was found at 12 months; again, close to 10 times less than in our study. However, their study included only 15 patients, making it somewhat underpowered. Hoornenborg et al. (2018) reported the migration and clinical outcome of the Zweymüller with or without HA coating and found a Y-translation of –0.46 mm (CI –0.73 to –0.19) with HA coating and –0.73 mm (CI –1.18 to –0.29) without HA coating. It has been estimated by Kärrholm et al. (1997) that subsidence of cemented stems should be less than 1.5 mm at 24 months; however, in recent years it has been proposed that for uncemented stems it is not the exact value of the subsidence, but instead the migration pattern, that anticipates the fate of the arthroplasty (Weber et al. 2014, Critchley et al. 2020). Accordingly, if a pattern of early subsidence is followed by stabilization at 3 months, one should not worry about stem failure. This seems to be true for our cohort. A possible explanation for the level of subsidence in our study might be the fear of creating a fissure or fracture by excessively forcing the stem into the femoral canal, combined with the collarless design of both stems. To investigate whether the surgeons could have tended to undersize the femoral components, a post hoc evaluation of each templated radiograph compared with the actual stem size together with a classification of Dorr type was performed (Ashraf 2018). Only in 1 case was a stem selected with a smaller size than templated (templated to EBM 13, actual EBM 12). No femurs were Dorr type C.

We had 3 outliers with retroversion (Y-rotation), up to approximately 14° in participant number 6 (Table 3). All 3 scored maximum points (or close to) in the clinical outcome measurements at 24 months.

Number 6 is an otherwise healthy man with a BMI of 25 and he received a standard EBM stem size 12. When reviewing and comparing the regular radiographs postoperatively (before mobilization) with the RSA about 1 week later we could see subsidence with the naked eye. It seems the stem subsided and rotated at mobilization and the stem chosen might have been too small. This had no clinical consequences; he had “no complaints at all” during a telephone call just before submission of this manuscript (approximately 3 years postoperatively). In patient number 8 there is also a visible difference between the regular radiographs before mobilization and the first RSA, but this is not the case with number 23.

Concerning the varus–valgus tilt (Z-rotation), normally stems migrate into a slight varus position. However, surprisingly we found the stems going into valgus. A possible explanation could be too extensive preparation of the trochanter major before insertion of the stem. This could also explain some of the subsidence. However, we have no obvious explanation for this phenomenon.

Clinical outcomes were satisfying with scores very close to the maximum for both groups at 24 months. The study by Klein et al. (2019), in which the Corail stem was evaluated prospectively, reports the median (range) HHS at 24 months as 100 (44–100) and the median (range) for OHS at 24 months as 45 (15–48), which is compatible with our results.

Our precision error, measured by double examinations, was comparable to the Nebergall et al. (2016) study. Nevertheless, we find there is room for improvement and this could be explained by our manual set-up with no automatic setting of X-ray tubes or fixed position of the calibration cage. To reduce bias, only the primary investigator handled the RSA set-up.

A limitation of our study might be the use of MB-RSA (Nazari-Farsani et al. 2016) and a phantom trial to determine the accuracy of our MB-RSA compared with marker-based RSA would have been ideal. Still, the advantage of not having to produce new adjusted stems with attached markers, and in some way changing the design, is desirable.

Another limitation of this study is the rather small sample size. Although we included 11 more individuals than estimated by the power calculation it is not a large cohort and a non-inferiority/equality study would be interesting. However, this would require an even larger cohort and is a slow process for a single hospital.

A certain level of selection bias might have reduced the quality of data, as the patients who accept to participate in this type of study are probably the most resourceful of all eligible, in the sense that they have accepted extra follow-ups with the hassle this entails. The generalizability of our findings is rather narrow because the results apply only to the population of otherwise healthy people who need a THA.

In conclusion, our hypothesis that the EBM stem migrates less than the BM stem was rejected. Thus, the added design features do not seem to have any consequences concerning stem migration or clinical outcome, suggesting negligible differences.

The authors thank the hip surgeons, J. Retpen, A. G. Kjersgaard and M. Skettrup, who, besides co-author S. Solgaard, agreed to perform the THA surgery according to the protocol.

They thank the surgical staff at Gentofte Hospital and chief radiographer Torben Kragelund and radiographer Mohammad Khonbat Lauritsen at the department of diagnostic radiology, Rigshospitalet. Finally, they thank Håkan Leijon of the RSA laboratory in Lund, Skåne University Hospital.
